# *Aporrectodea caliginosa*, a relevant earthworm species for a posteriori pesticide risk assessment: current knowledge and recommendations for culture and experimental design

**DOI:** 10.1007/s11356-018-2579-9

**Published:** 2018-06-21

**Authors:** Sylvain Bart, Joël Amossé, Christopher N. Lowe, Christian Mougin, Alexandre R. R. Péry, Céline Pelosi

**Affiliations:** 1 UMR1402 ECOSYS, INRA, AgroParisTech, Université Paris-Saclay, Bâtiment 6 RD 10, 78026 Versailles Cedex, France; 2 School of Forensic and Applied Sciences, University of Central Lancashire, Preston, PR1 2HE UK

**Keywords:** *Aporrectodea caliginosa*, Plant protection products, Soil ecotoxicology, Agroecotoxicology, *Lumbricidae*, Agroecosystems, Breeding

## Abstract

Ecotoxicological tests with earthworms are widely used and are mandatory for the risk assessment of pesticides prior to registration and commercial use. The current model species for standardized tests is *Eisenia fetida* or *Eisenia andrei.* However, these species are absent from agricultural soils and often less sensitive to pesticides than other earthworm species found in mineral soils. To move towards a better assessment of pesticide effects on non-target organisms, there is a need to perform a posteriori tests using relevant species. The endogeic species *Aporrectodea caliginosa* (Savigny, 1826) is representative of cultivated fields in temperate regions and is suggested as a relevant model test species. After providing information on its taxonomy, biology, and ecology, we reviewed current knowledge concerning its sensitivity towards pesticides. Moreover, we highlighted research gaps and promising perspectives. Finally, advice and recommendations are given for the establishment of laboratory cultures and experiments using this soil-dwelling earthworm species.

**Electronic supplementary material:**

The online version of this article (10.1007/s11356-018-2579-9) contains supplementary material, which is available to authorized users.

## Introduction

The use of pesticides may harm soil fauna, which are involved in key soil functions and related ecosystem services (McLaughlin and Mineau 1995; Blouin et al. 2013; Bertrand et al. 2015). Earthworms are ecosystem engineers (Jones et al. 1994), representing the most important living biomass in terrestrial ecosystems, often up to one ton per hectare in hardwood forests and in pastures (Lavelle and Spain 2001). They modify soil structure and improve water regulation, nutrient cycling, and primary production (Lavelle et al. 2004; Seeber et al. 2008; Bottinelli et al. 2010; Blouin et al. 2013). Moreover, they are recognized as indicators of soil biological activity (Paoletti 1999) and have been used as model organisms in soil ecotoxicology for more than 30 years (OECD 1984; Spurgeon et al. 2003).

During the 1980s, standardized acute tests with earthworms were developed to assess the effects of pollutants (OECD 1984). Subsequently, other standardized tests, assessing earthworm survival, reproduction, and behavior, were approved by the International Organization for Standardization (ISO) (ISO 2008, 2012a, b) and/or by the Organization for Economic Co-operation and Development (OCDE 2004). These tests are regularly updated and the OECD guideline 222 (2004) is used in the risk assessment process associated with the registration of new pesticides (EFSA 2017). *Eisenia fetida* (Savigny, 1826) or *Eisenia andrei* Bouché (1972) are recommended as test species in standardized tests and are widely used in ecotoxicological studies because they are relatively easy to breed and have a short generation time (OECD 1984), allowing for quick and cost-effective tests. Despite these advantages, these species generally do not inhabit mineral soils (Lowe and Butt 2007) and therefore are rarely found in cultivated fields where pesticides are applied. Furthermore, Pelosi et al. (2013) highlighted that LC50 values for *Lumbricus terrestris* (Linnaeus, 1758) and *Aporrectodea caliginosa* (Savigny, 1826), two common soil-dwelling species in cultivated fields, were significantly lower than LC50 values for *E. fetida.* Consequently, these authors recommended *A. caliginosa* to be used in ecotoxicological tests. Similarly, van Capelle et al. (2016) proposed *A. caliginosa* and *L. terrestris* as non-target soil organisms for environmental risk assessment of genetically modified plants. Klobucar et al. (2011) also proposed *A. caliginosa* for genotoxicity field surveys using biomarkers. The use of *A. caliginosa* species to test the effects of pesticides is thus increasingly argued in the scientific literature (Booth and O’Halloran 2001; Spurgeon et al. 2003). Other temperate soil-dwelling species such as *Lumbricus rubellus* Hoffmeister, 1843 or *Octolasion cyaneum* (Savigny, 1826) have also been advocated for ecotoxicological tests (Lowe and Butt 2007). However, *L. rubellus* being epi-endogeic is rarely found in conventionally cultivated fields due to the lack of surface litter and Pelosi et al. (2013) showed that the sensitivity of *L. rubellus* to pesticides was lower than that of *E. fetida*. The endogeic species *O. cyaneum* is widespread but not common in all cultivated fields because it prefers moist habitats such as wet sands and reproduces parthenogenetically (Sims and Gerard 1999).

**Fig. 1:**
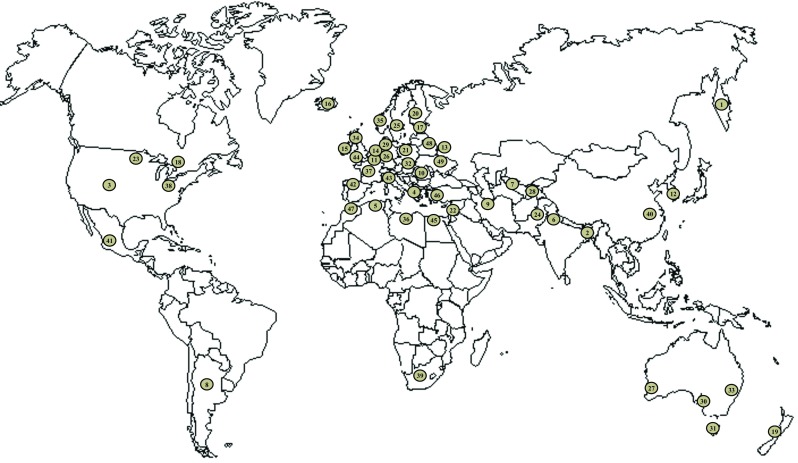
World distribution map of *A. caliginosa* based on the literature review. Each number represents an area or a country where the species was found (see Appendix, Table S1 for the list of countries or regions and references)

**Fig. 2:**
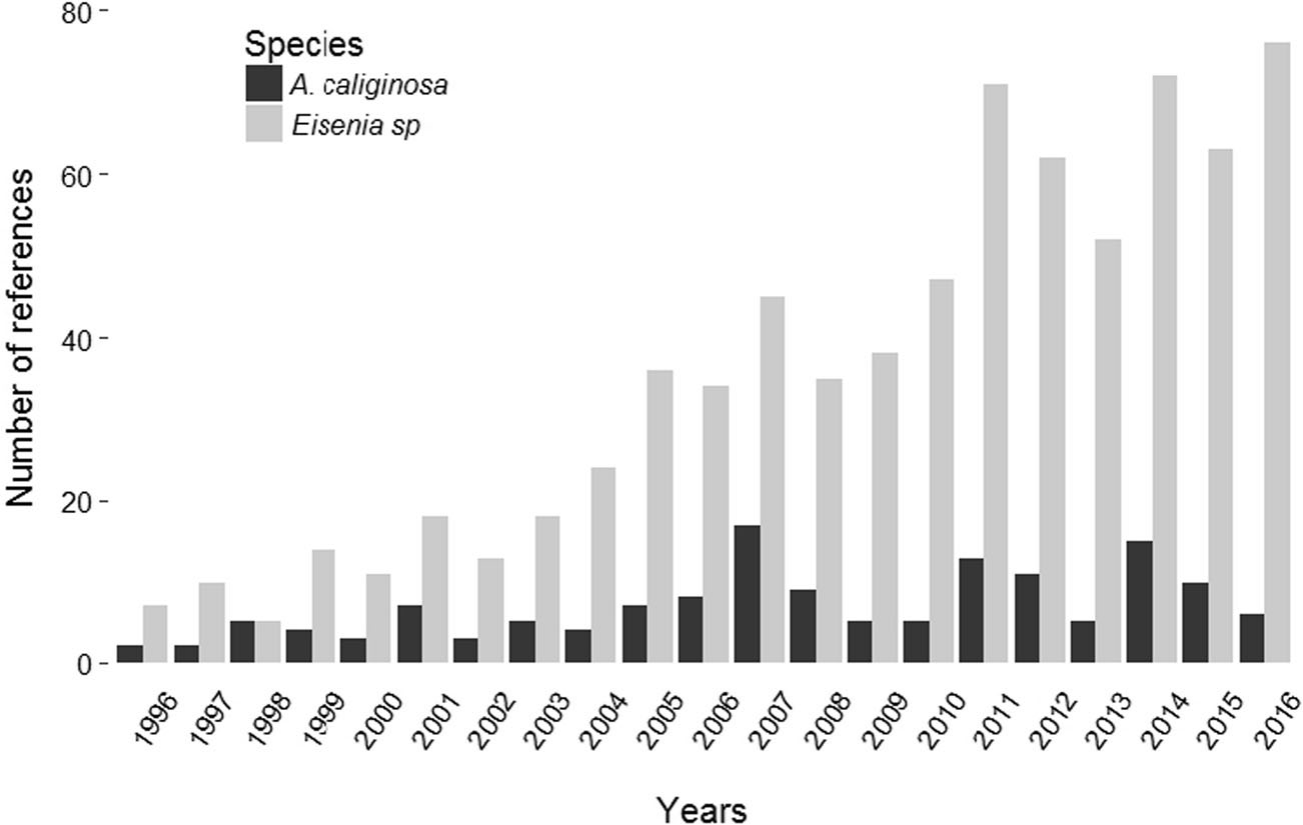
Number of references dealing with *Aporrectodea caliginosa* and *Eisenia* sp. and pesticides over the last 20 years (Source: ISI Web of Knowledge, using “All Databases” option, with the formula (in Topic): (pesticid* OR herbicid* OR fungicid* OR molluscicid* OR nematicid* OR insecticid* OR plant protection product* OR crop protection product*) AND (eisenia OR fetida OR foetida OR andrei) for *Eisenia* sp. or (caliginosa* AND earthworm*) for *A. caliginosa*. The search was performed in 2017 and includes references from 1996 to 2016

The endogeic species *A. caliginosa* is ubiquitous and data obtained from the reviewed literature was used to map its global geographical distribution (Fig. 1). We recorded all the countries or regions where *A. caliginosa* were sampled and the study purpose (i.e., ecology, ecotoxicology, or biodiversity studies). *A. caliginosa* has been found in all temperate zones (i.e., in Europe, America, Asia, Oceania, and South Africa, see Fig. 1). Moreover, it is one of the most abundant species in most soils (and in particular cultivated soils) of temperate zones (Boström and Lofs-Holmin 1996; Boag et al. 1997; Curry et al. 2008; Perez-Losada et al. 2009; Decaëns et al. 2011). It lives in the first 15 cm of soil and is highly representative of agricultural soils, which is one of the two most relevant criteria for test organisms (i.e., the representativeness of the ecosystem to protect) according to the European Food Safety Authority (EFSA 2017). *A. caliginosa* displays high ecological plasticity and adaptability in agroecosystems (Bouché 1972; Sims and Gerard 1999), especially to agricultural practices such as soil tillage (Crittenden et al. 2014). In addition, *A. caliginosa* is able to survive in soils with low organic matter (1.4% organic carbon, McDaniel et al. 2013) and moisture content (at least 3 weeks under drought conditions, McDaniel et al. 2013). *A. caliginosa* plays several key ecological roles such as nutrient cycling (e.g., increasing nitrogen flux and lowering the C/N ratio (Sandor and Schrader 2007; McDaniel et al. 2013) and enhancing nutrient availability for plants and microorganisms (Sharpley and Syers 1976; Sharpley and Syers 1977). *A. caliginosa* can also increase microbial biomass (Svensson and Friberg 2007) and its relatively high burrowing activity can have a positive impact on water infiltration /discharge (Ernst et al. 2009; McDaniel et al. 2015) and on soil aeration (Francis and Fraser 1998). Nevertheless, *Eisenia* sp*.* remains the most frequently used species in assessing the impact of applied pesticides on earthworms (Fig. 2). In 2016, 76 references involving pesticide effects on *E. fetida* were found compared with only 6 for *A. caliginosa* (Fig. 2), probably due to technical difficulties involved in obtaining a sufficient number of individuals for experiments. In addition, it is recognized that *A. caliginosa* has wide ranging morphological variation as it is a complex of species (see section Taxonomic considerations). This issue, addressed in this paper, can hinder the use of this species as a laboratory test organism. In this paper, we focused on *Aporrectodea caliginosa* s.s, commonly recognized as a separate species (Sims and Gerard 1999) and often referred to in current scientific literature as *A. caliginosa*.

We aimed to review current knowledge about *A. caliginosa* in order to assess its suitability as a species for a posteriori pesticide risk assessment, and to give recommendations for laboratory culture and experimental design. General information on the biology and the ecology of this species (i.e., taxonomic aspects, morphological description, geographical distribution, and life history traits) are provided and the effects of pesticides on its life history traits and behavior are then reviewed. Finally, based on published information and on our own experience, advice on the establishment of laboratory cultures and experiments are provided. The study highlights knowledge gaps, further research opportunities, and perspectives to promote the use of *A. caliginosa* as a suitable species in a posteriori pesticide risk assessment procedures and for research in soil ecotoxicology.

## Search method

A systematic literature review was conducted based on keywords in the ISI Web of Knowledge, using the “All Databases” option, with the following formula: caliginos* AND earthworm* in Topics. One thousand one hundred ninety-five references were obtained. This review focused on the endogeic *A. caliginosa* which can be found in literature under different names (see section Taxonomic considerations). Articles referring to *Aporrectodea tuberculata*, *Aporrectodea nocturna*, and *Aporrectodea trapezoides* were excluded*.* References related with *A. caliginosa* biology and ecology as well as the impacts of pesticide and metal contaminants on life history traits and behavior were selected. Literature associated with impacts of heavy metals on *A. caliginosa* under laboratory conditions are presented in Table S2 but they have not been incorporated into this review because they were not directly associated with pesticide risk assessment. For the purposes of this study, pesticides are defined as chemicals used against “harmful” organisms in agroecosystems. The titles were first read, then a second selection was made based on the abstracts and finally on full texts. Thus, 27 publications, related to the impact of organic pesticides on life history traits and behavior of *A. caliginosa*, were selected (see the “Sensitivity to pesticides” section). Finally, selected references on the biology and ecology of *A. caliginosa* were used to inform other parts of this review (see the “Morphology and life history traits” and “Laboratory culture and experimental design” sections).

## Taxonomic considerations

*Aporrectodea caliginosa* (Savigny, 1826) belongs to *Annelida: Oligochaeta: Lumbricidae.* Taxonomic classification has changed several times and it is considered a species complex. Bouché (1972) divided *A. caliginosa* into two species with another genus name: *Nicodrilus caliginosus* (composed of three subspecies: *N. c. caliginosus*, *N. c. alternisetosus*, and *N. c. meridionalis*) and *Nicodrilus nocturnus*). Sims and Gerard (1985) reported that four morphs were commonly recognized as separate species: *A. caliginosa* s.s (syn. *turgida*), *A. tuberculata*, *A. nocturna*, and *A. trapezoides.* The variations in these four species are now considered to be mostly phenotypic. In this paper, we refer exclusively to the *A. caliginosa* s.s morph which can be considered as a “morphospecies” (i.e., a species distinguished from others only by its morphology). More recently, Briones (1996) proposed two subspecies: *A. caliginosa caliginosa* and *A. caliginosa trapezoides* but Perez-Losada et al. (2009) and Fernández et al. (2012) have shown that *A. caliginosa* and *A. trapezoides* are two clearly different species and that *A. caliginosa* is closer to *A. tuberculata*. To summarize, information on *Aporrectodea caliginosa* presented in this review was recorded under different names in the scientific literature: *Aporrectodea caliginosa* s.s., *Aporrectodea caliginosa caliginosa* (or *Allolobophora caliginosa caliginosa*), and *Nicodrilus caliginosus caliginosus*. This endogeic species is currently named *Aporrectodea caliginosa* and is referred to as such in the rest of this paper.

Diaz Cosin et al. (2011) explained that comparing published data on species that belong to a complex of species could be hazardous, as the authors may have incorrectly identified the species. This is the case for *A. caliginosa* but it could be the same for other species, e.g., Cunha et al. (2014) revealed the existence of cryptic lineages (morphospecies) within *Pontoscolex corethrurus* using molecular markers. Even *E. fetida* and *E. andrei*, used as model species in ecotoxicology, are part of a species complex (Latif et al. 2017).

In this situation, it is proposed that a detailed description of individuals along with geographic origin should be provided in any associated publications. Moreover, when possible, individuals should be DNA barcoded to verify if they belong to *A. caliginosa*.

## Morphology and life history traits

Adults of *A. caliginosa* are typically composed of 120–150 segments. They range in length from 60 to 85 mm, with a biomass of 200 to 1200 mg (Sims and Gerard 1999). Individuals lack any significant pigmentation but the anterior segments are pale pink in coloration (Sims and Gerard 1999; Bouché 1972). Reproduction is obligatory biparental and the saddle-shaped clitellum extends over at least six segments (27) 31–34 (35) (Sims and Gerard 1999). The tubercula pubertatis is ridge-like over segments 31–33 and often bipartite, divided by a transverse furrow on segment 32.

**Fig. 3:**
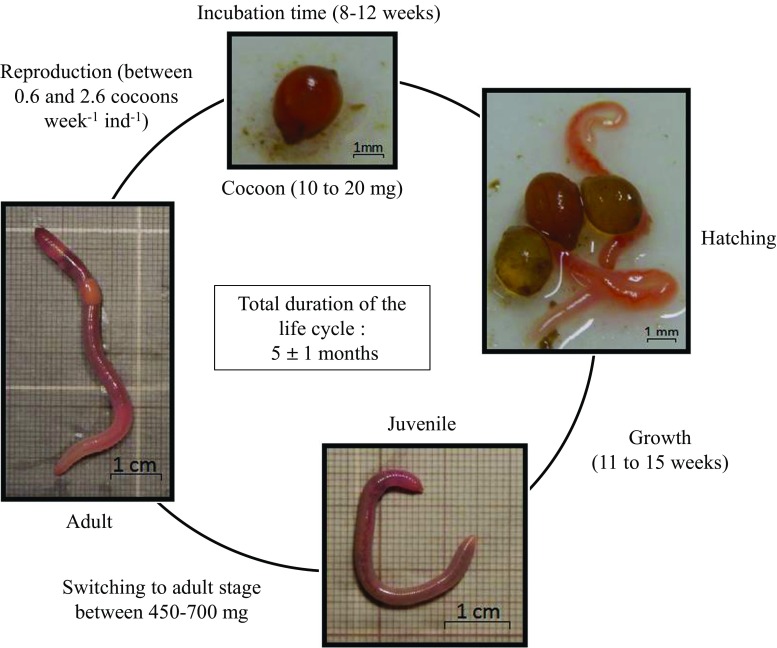
Life cycle of *A. caliginosa* under laboratory conditions in a loamy field soil supplemented with horse dung milled at 1 mm as food, 15 °C, 60–70% of the soil water holding capacity (personal observations, photos ©Sylvain Bart)

The life cycle of *A. caliginosa* under laboratory conditions is described in Fig. 3. Information on cocoon production, incubation time and viability are summarized by Lowe and Butt (2005). According to this review and other articles (e.g., Lofs-Holmin 1982; Spurgeon et al. 2000), an individual *A. caliginosa* can produce between 0.6 and 2.6 cocoons per week at 15 °C in a field soil. Fecundity not only depends on environmental, physical, and chemical soil characteristics but also on the age because individuals may suffer reproduction fatigue. In petri dishes on a moist filter paper, Holmstrup et al. (1991) recorded cocoon incubation periods of 36, 62, 199, and 234 days at 20, 15, 10, and 5 °C, respectively. Other authors reported incubation periods of 56–63 days (Jensen and Holmstrup 1997) at 15 °C in petri dishes or 70–84 days at 15 °C in soil (Boström and Lofs-Holmin 1996). The hatching rate (i.e., cocoon viability) was found to vary between 90 and 97% at 15 °C in petri dishes (Holmstrup 2001; Jensen and Holmstrup 1997). Similarly, Boström and Lofs-Holmin (1996) recorded a hatching rate of 91–95% at 15 °C in a field soil, but Booth et al. (2000a) reported a rate of 61 and 80% after 28 and 56 days respectively at 20 °C in petri dishes on moist filter paper. Booth et al. (2000a) found that 75 to 80% of individuals became adult after 18 weeks at 15 °C. Lofs-Holmin (1983) found a shorter maturation time of 6.5 weeks at the same temperature in a mixture of farmyard manure and clay. From our own observations, under laboratory conditions (i.e., field loamy soil with horse dung as food, 15 °C, 60–70% soil Water Holding Capacity (WHC)), this species needs an average of 13 ± 2 weeks to mature from hatchling emergence. Cocoon biomass ranges from 10 to 20 mg and juveniles generally become adults at a biomass of 450 to 700 mg. To summarize the literature and our own observations, under laboratory conditions at 15 °C, the life cycle duration of *A. caliginosa* is between 4 and 6 months (Fig. 3). The life span is unknown but is regularly more than 2 years under laboratory conditions (personal observation).

## Sensitivity to pesticides

Twenty-seven publications dealing with the impacts of pesticides on life history traits and behavior of *A. caliginosa* are summarized in Table 1*.* In 15 studies, authors used commercial formulations of pesticides to allow better understanding and assessment of ecotoxicological effects under field conditions and 6 studies used pure molecules (6 studies did not report the form). Twenty-three publications assessed the impacts of insecticides while only 7 dealt with herbicides and 4 with fungicides (some articles assessed different types of pesticides). Since herbicides and fungicides represent the main pesticides applied in agroecosystems (respectively 45 and 37% of the market in France in 2015 (UIPP 2016)), further research is needed to assess the ecotoxicological effects of these products on *A. caliginosa.*

**Table 1:** Effects of pesticides on life history traits and behavior of *A. caliginosa* under laboratory conditions

Reference	Studied parameters	Origin	Development stage	Pesticide used	Active substance	Action	Method of addition	Duration	Main results
Alshawish et al. (2004)	Biomass and cocoon hatching	NA	NA	NA	Chlorpyrifos Cypermethrin Mancozeb	Insecticide Insecticide Fungicide	Mixed	12 weeks	Higher body mass increase in the control. Toxicity of chlorpyrifos (100% hatching failure) > mancozeb (> 73% hatching failure) > cypermethrin (80% survival and normal hatching).
Badawy et al. (2013)	Mortality and biomass	Collected	Adult	F	Buprofezin Triflumuron Lufenuron	Insecticide Insecticide Insecticide	NA	28 days	LC50 lufenuron: 1.87 mg/kg > LC50 buprofezin: 421 mg/kg > LC50 triflumuron: 477 mg/kg. The reduction in biomass was dose-dependent in all treatments.
Bart et al. (2017)	Mortality, biomass and avoidance	Collected	Adult	F	Dimoxystrobin and epoxiconazole	Fungicide	Mixed	14 days for the acute test, 48H for the avoidance test	LC50: 6.3 times the RD. No avoidance from 1 to 10 times the RD. No biomass loss.
Booth et al. (2000a)	Growth, cocoon production, hatching success	NA	Juvenile	F	Chlorpyrifos Diazinon	Insecticide Insecticide	Mixed	4 weeks	For both insecticides: at the RD, no significant effect on growth, cocoon production and hatching success. At 5 times the RD for diazinon and 7 times for chlorpyrifos, there was a decrease in biomass (but reversible after 8 weeks without pesticide). A slow maturation and a reduction in the cocoon production was observed only for chlorpyrifos at 7 times the RD.
Booth et al. (2000b)	Mortality	Cultured	Juvenile	F	Chlorpyrifos Diazinon	Insecticide Insecticide	Mixed	14 days	LC50 chlorpyrifos: 69 mg/kg, LC50 diazinon: 100 mg/kg.
Booth and O’Halloran (2001)	Growth, maturation, cocoon production, and viability	Cultured	Adult and juvenile	F	Chlorpyrifos Diazinon	Insecticide Insecticide	Mixed	12 weeks	At 7 times the RD of chlorpyrifos and from the RD of diazinon, negative effects on growth, maturation, and cocoon production. The maturation was less sensitive than cocoon production. Growth and cocoon production in earthworms exposed as juveniles were more sensitive than earthworms exposed as adult.
Dalby et al. (1995)	Mortality and biomass	Collected	NA	NA	Dimethoate Glyphosate 2,4-DB	Insecticide and Acaricide Herbicide Herbicide	Sprayed	10 days or 3 weeks	No effects on survival or biomass, with or without plant cover.
Dittbrenner et al. (2010)	Biomass and cast production	Collected	Adult	M	Imidacloprid	Insecticide	Mixed	7 days	Significant biomass loss from the RD (0.66 mg/kg) to 4 mg/kg. Decrease in cast production at 0.66 and 2 mg/kg.
Dittbrenner et al. (2011a)	Burrowing behavior	Collected	Adult	M	Imidacloprid	Insecticide	Mixed	1, 7, or 14 days, 24–96 h for short-term effects and 6 weeks for long-term effects	Negative effects for the short-term experiment on the burrow depth from 0.2 to 4 mg/kg. Significant linear decrease in burrow volume with increasing imidacloprid concentration. RD = 0.66 mg/kg.
Dittbrenner et al. (2011b)	Biomass	Collected	Adult	M	Imidacloprid	Insecticide	Mixed	7 and 14 days	Significant biomass loss from 0.2 to 4 mg/kg after 7 days and from 0.66 to 4 mg/kg after 14 days. RD = 0.66 mg/kg.
Dittbrenner et al. (2012)	Avoidance behavior	Collected	Adult	M	Imidacloprid	Insecticide	Mixed	48 h	No avoidance of imidacloprid-contamined soil.
Falco and Momo (1991)	Mortality, biomass, cocoon production and fecal pellets production.	NA	Adult	NA	Heptachlor	Insecticide	NA	8 days	At 1.69 mg/kg, mortality increased with the average mass of earthworms. The decrease in mean biomass was maximal for individuals with higher average mass and minimal for individuals with an intermediate mass. No effect on cocoon production or fecal pellet production.
Gaupp-Berghausen et al. (2015)	Cocoon hatching, surface casting activity	Collected	Adult/Sub-adult	F	Glyphosate	Herbicide	Sprayed	4 weeks for casting activity and 3 months for cocoon hatching	At 176.12 mL/m − 2 of herbicide, which is 53% lower than the recommended plant-based application rate, reproduction was reduced by 56% within 3 months after herbicide application. Casting activity was not affected.
Hodge et al. (2000)	Avoidance behavior	NA	Juvenile	F	Chlorpyrifos Diazinon	Insecticide Insecticide	Sprayed	1 or 4 days	No avoidance.
Kula and Kokta (1992)	Mortality	Collected	Adult	F	Parathion Propoxur	Insecticide Insecticide	Mixed	14 days	LC50 parathion: 126 mg/kg and LC50 propoxur: 4.5 mg/kg.
Kula and Larink (1997)	Mortality	Collected	Adult and juvenile	NA	Dimethoate	Insecticide and Acaricide	Mixed	28 days	LC50: 179 and 10 mg/kg for adults and juveniles respectively in OECD soil, and 47 and 32 mg/kg for adults and juveniles in LUFA soil, respectively.
Ma and Bodt (1993)	Mortality	Collected	Adult	M	Chlorpyrifos	Insecticide	NA	14 days	LC50: 755 mg/kg, NOEC: 486 mg/kg.
Martin (1986)	Mortality, biomass and cocoon production	Collected and Cultured	Adult and juvenile	F	Carbofuran Aldicarb Phorate Aldoxycarb Methomyl Oxamyl Isazophos Fenamifos Fensulfothion Diazinon Citowett	Insecticide Insecticide Insecticide Insecticide Insecticide Insecticide Insecticide Insecticide Insecticide Insecticide Herbicide		7 days for juvenile and 14 days for mature individuals	Concentration (mg/kg of dry soil) of pesticide causing zero growth (compared with the standard): carbofuran (0.10), aldicarb (0.09), phorate (0.30), aldoxycarb (0.40), methomyl (0.54), oxamyl (0.59), isazophos (0.93), fenamifos (5.72), fensulfothion (7.35), diazinon (12.4), and citowett (525). For mature individuals, 0.5, 1 and 5 mg/kg of carbofuran caused 10, 80 and 100% of mortality respectively. These concentrations also reduced cocoon production.
Mosleh et al. (2003)	Mortality and biomass	Collected	Adult	F	Aldicarb Cypermethrin Profenofos Chlorfluazuron Atrazine Metalaxyl	Insecticide Insecticide Insecticide Insecticide Herbicide Fungicide	Mixed	7, 14, 21 and 28 days	LC50 after 28 days of exposure was 0.68, 72.96, 127.00, 139.90, 381.20 and 518.00 mg/kg for aldicarb, cypermethrin, profenofos, chlorfluazuron, atrazine and metalaxyl respectively. There was a decrease in biomass at the LC25 for all these pesticides.
Mosleh (2009)	Mortality	Collected	Adult	NA	Isoproturon	Herbicide	Mixed	60 days	No mortality. LC50 > 1200 mg/kg
Olvera-Velona et al. (2008)	Mortality, biomass and burrowing behavior	Collected	Adult	F	Ethyl-parathion	Insecticide	Mixed	7 and 14 days	Mortality occured at lower concentrations after 14 days than 7 days. LC50 between 11 to 57 times RD, depending on the soil and time of exposure (7 or 14 days). Body mass change was significantly affected by pesticide concentration and in a lower extent by soil type (since 10 x RD). Reduction in burrow length and number of branches from 1 time the RD.
Rault et al. (2008)	Biomass	Collected	Adult	F	Ethyl-parathion	Insecticide	Mixed	14 days exposure and 56 days recovery	At 10 mg/kg, after 28 days, significant biomass loss. At 1 mg/kg, after 3 days, decrease in biomass but *A. caliginosa* showed rapid mass recovery.
Reinecke and Nash (1984)	Mortality, body observation	NA	Juvenile	NA	Dioxin	Herbicide	NA	85 days	No worms died or showing any other observable toxicological effects when exposed to concentrations up to 5 mg/kg. The lethal threshold concentration was between 5 and 10 mg/kg.
Reinecke and Reinecke (2007a)	Biomass	Collected	Adult	F	Chlorpyrifos	Insecticide	Sprayed	2 weeks	Significant decrease in biomass at the commercially recommended rate.
Reinecke and Reinecke (2007b)	Biomass, state of estivation	Collected	Adult	F	Chlorpyrifos	Insecticide	Sprayed	5 weeks	The highest biomass loss was observed with earthworms exposed to the highest pesticide concentration (8 μg/kg). Estivation was higher among earthworms exposed to higher exposure concentrations.
Springett and Gray (1992)	Growth, maturation and mortality	Cultured	Juvenile	F	Captan Glyphosate Azinphos-methyl	Fungicide Herbicide Insecticide	Sprayed	100 days	Azinphos-methyl and glyphosate reduced growth at all the concentration (realistic field rates). Captan had the lowest effect on growth and mortality. Worms took more time to reach maturity in all the other treatments except with Captan at the mid rate.
Stenersen (1979)	Mortality	Collected	Adult	M	Aldicarb Carbaryl Carbofuran Oxamyl Paraoxon Parathion Trichloronate gamma-HCH	Insecticide Insecticide Insecticide Insecticide Insecticide Insecticide Insecticide Insecticide	Mixed	14 days in soil and 30 min in pesticide solution	The range of LC50 (pesticide solution) were 3.1–6.3, > 200, 3.1–6.3, 0.39–0.78 and 400–800 μg/mL for aldicarb, oxamyl, carbaryl, carborufan and Gamma-HCH respectively. Carbaryl and carbofuran were lethal in soil at low concentration (4 mg/kg).

For origin: cultured (in laboratory) or collected (in the field). Pesticide used: F = formulation and M = pure molecule. Method of addition: mixed (into the soil) and sprayed (at soil surface). *RD* recommended dose, *LC50* lethal concentration for 50% of exposed individuals, *NOEC* no observed effect concentration, *NA* not available, *EC*_*X*_ effective concentration

Fifteen publications presented data on mortality to estimate LC50 values. These values, which are commonly used in ecotoxicological studies, have been used to compare sensitivity between species and taxa (Pelosi et al. 2014; Bart et al. 2017). In most studies, LC50 was estimated using adults but a few studies have also compared these values for both adults and juveniles. Kula and Larink (1997) assessed the effects of the insecticide dimethoate on *A. caliginosa* in OECD artificial soil (10% sphagnum, 20% kaolin clay, 70% sand) and estimated LC50 at 179 mg kg^−1^ for adults and 10 mg kg^−1^ for juveniles. With the same pesticide in a LUFA 2.2 soil (Speyer, Germany) (standardized soil composed of 6.7% clay, 15.4% silt and 78.1% sand), an LC50 of 47 and 32 mg kg^−1^ was recorded for adults and juveniles, respectively. This could be due to the higher availability of the contaminant in LUFA soil. For the insecticide chlorpyrifos, LC50 was estimated to be 755 mg kg^−1^ for adult individuals (Ma and Bodt 1993) and 69 mg kg^−1^ for juveniles (1–3 months old) (Booth et al. 2000b). Although these two studies were not undertaken in the same conditions (e.g., soil, temperature), juveniles appeared to be more sensitive to pesticides than adults. Several authors have reported the same findings for other earthworm species (e.g., *Eisenia fetida andrei* by Zhou et al. (2008)). Therefore, acute toxicity of pesticides to juveniles should be more widely examined to better understand and to assess agroecotoxicological effects of pesticides on earthworm life cycles.

In addition to acute studies, different sublethal (chronic) end points, including reproduction that is required for pesticide registration, have also been used. Firstly, fecundity (cocoon production and/or viability) was studied in 6 out of 27 articles. Booth et al. (2000a) found a reduction in cocoon production at 7 times the recommended dose (RD) of chlorpyrifos (28 mg kg^−1^). Similarly, Gaupp-Berghausen et al. (2015) showed that glyphosate reduced hatching rate by 56% at half of the RD. In addition, Booth and O’Halloran (2001) found that juveniles exposed to realistic field applications of chlorpyrifos (4 mg kg^−1^) and diazinon (12 mg kg^−1^) produced subsequently fewer cocoons when they became adults than mature earthworms exposed to the same concentrations of these pesticides. These results suggested that (i) the reproduction of *A. caliginosa* individuals can be affected by pesticides at realistic concentrations and (ii) pesticides are more harmful to juveniles than adults. This may have consequences for life cycle and population dynamics of *A. caliginosa* in natural conditions.

Some authors used the term “growth” to describe biomass change of adult individuals (Dalby et al. 1995; Mosleh et al. 2003; Badawy et al. 2013). From our point of view, “growth” should be restricted to juveniles and we recommend use of the terms “biomass,” gain or loss, or “weight change” for adult individuals. Biomass monitoring of adult individuals was used in 13 out of 27 studies and provided useful information on earthworm health at low pesticide concentrations. For example, Reinecke and Reinecke (2007a) and Dittbrenner et al. (2011b) showed a significant decrease in biomass at realistic concentrations of chlorpyrifos and imidacloprid respectively.

Data on the influence of pesticides on growth and maturation (defined by the presence of a fully developed clitellum) were only found in three publications. Booth et al. (2000a) showed that the growth of *A. caliginosa* was significantly reduced by chlorpyrifos at the RD and by diazinon at five times the RD. However, there was no effect during the recovery period (i.e., period in a control soil after exposure to a contaminated soil). In this study, juvenile individuals exposed to seven times the RD of chlorpyrifos and five times the RD of diazinon matured more slowly than earthworms in control soil without pesticides. It has also been reported that the herbicide glyphosate and the insecticide azinphos-methyl repeatedly applied at 2-weekly intervals at lower concentrations than the commercial RD decreased growth rates and increased time to reach maturity of *A. caliginosa* (Springett and Gray 1992). However, these studies were performed with juveniles of unknown ages. In this study and more generally, the authors did not assess the impact of pesticides on earthworm growth from hatching to maturity. In all related studies, only the biomass of juveniles was given and was at least 200 mg at the beginning of the experiment compared to the biomass of *A. caliginosa* individuals just after hatching which is between 10 to 30 mg (Lofs-Holmin 1983; Boström and Lofs-Holmin 1986; Boström 1988). It would therefore be relevant for the assessment of pesticide impacts to assess effects on recently hatched individuals. For that, cohorts of recently hatched individuals can be maintained at 4 °C to slow growth rates (see the “Laboratory culture and experimental design” section).

Finally, *A. caliginosa* behavior in response to pesticide exposure can be assessed using avoidance, casting or burrowing activity. At three times the RD of imidacloprid (Dittbrenner et al. 2012), 16 times the RD chlorpyrifos (i.e., Lorsban 40EC, Hodge et al. 2000), and 10 times the RD of dimoxystrobin and epoxiconazole (i.e., Swing® Gold, Bart et al. 2017), no avoidance was reported. Although these results give some indication of earthworm behavior in response to pesticide application in field soils, they are not sufficient to generalize on the capability of *A. caliginosa* to avoid pesticides in soils and further studies are required. Moreover, more realistic assessment of avoidance behavior may be assessed in a linear pollution gradient as proposed by in Lowe et al. (2016) and demonstrated in Brami et al. (2017b).

Five publications were found on casting and burrowing activity that can be related to ecological functions provided by *A. caliginosa* under pesticide exposure. Burrowing activity is involved in soil aeration and water infiltration (Ernst et al. 2009) and cast production can be related to organic matter degradation (Frouz et al. 2011). Dittbrenner et al. (2011a) showed a decrease in burrow volume at the RD and with increasing concentrations of imidacloprid. Similarly, Olvera-Velona et al. (2008) found that the insecticide ethyl-parathion applied at the RD decreased burrow length and the number of branches in a Calcosol. Dittbrenner et al. (2010) showed a decrease in cast production of between 45 and 97% due to imidacloprid at an application rate of 0.66 mg kg^−1^ (1 times the RD).

To summarize, pesticides used at realistic field concentrations can induce sublethal negative effects on *A. caliginosa*. Chronic endpoints are useful to understand and predict pesticide impacts on the life cycle and population dynamics of this earthworm species. To go further, relationships between earthworm population dynamics and ecological functions should be assessed to allow an understanding and quantification of the impacts of pesticide application on ecosystem services provided by *A. caliginosa.* To reach this goal, there is a need for more knowledge and data on the impacts of pesticides on different developmental stages and ecological functions. However, it is difficult to get cohorts of sufficient number for robust and reliable experimentations. The next section provides information on overcoming this technical issue.

## Laboratory culture and experimental design

As discussed in the previous sections, there is a need to produce data on the impacts of pesticides on *A. caliginosa* life history traits, behavior and ecological function. A large number of individuals are thus required for these experiments. Earthworms can be collected from the field but retrieving a large number of individuals (> 200) is not always easy or feasible throughout the year. The collection of earthworms from the field is generally not possible during hot, cold and dry periods (i.e., during winter and summer). Furthermore, the age and exposure history of field-collected earthworms is unknown. Another solution is to culture *A. caliginosa* under controlled laboratory conditions*.* However, the procedure is poorly documented, except in Lowe and Butt (2005) who reviewed optimal laboratory conditions to be used for the culture of *A. caliginosa*. Based on their work, other publications and our own experience, we have summarized and carefully described the optimal conditions and steps for the establishment of a culture and the implementation of laboratory experiments using *A. caliginosa*.

Culture parameters are summarized in Table 2 (adapted from Lowe and Butt 2005). For maintenance and development of *A. caliginosa*, a field loamy/clay soil (pH 6–7) is more appropriate than standardized soils such as OECD and LUFA 2.2 soil (Brami et al. 2017a). Kula and Larink (1997) reported no cocoon production in a LUFA 2.2 soil (a sandy soil) due to earthworm inactivity. More generally, standardized soils, such as OECD soil, have been shown to be unsuitable for soil-dwelling species (Brami et al. 2017a). The soil needs to be pre-treated (air dried and sieved or crushed to 2–3 mm) to remove macro- and meso-invertebrates, and free from pesticides. Soil moisture must be adjusted to 25–30% (or 60–70% of the WHC), and the temperature must be around 15 °C (use of a temperature controlled room or incubator is advisable). The supplied food should be animal dung (cattle or horse dung is preferable), free from antibiotics/contamination, previously dried, milled (< 1 mm), rewetted and mixed into the soil. For optimal growth, it is suggested that 2–3 g and 4–6 g of dried food per individual per month for juveniles and adults respectively is provided.

**Table 2:** Guidelines for sustained culture of *A. caliginosa* (adapted from Lowe and Butt 2005)

Culture parameters	
Soil type	Natural loamy/clay soil (pre-treated to remove macro- and meso-invertebrates)
Soil depth (cm)	> 3 cm
pH	6–7
Soil moisture (%)	25–30% or 60–70% of the water holding capacity
Food	Dried and rewetted animal dung (cattle or horse)
Food amount for juveniles < 300 mg (ind^-1^ month^-1^)	2–3 g
Food amount for adults and juveniles > 300 mg (ind^-1^ month^-1^)	4–6 g
Food location	Mixed into the soil
Food particle size (mm)	< 1
Temperature (°C)	15 ± 1
Light	24 h dark
Vessel type	Sealed, opaque, preferably plastic with ventilation holes in the lid

### Development and maintenance of a laboratory culture

**Fig. 4:**
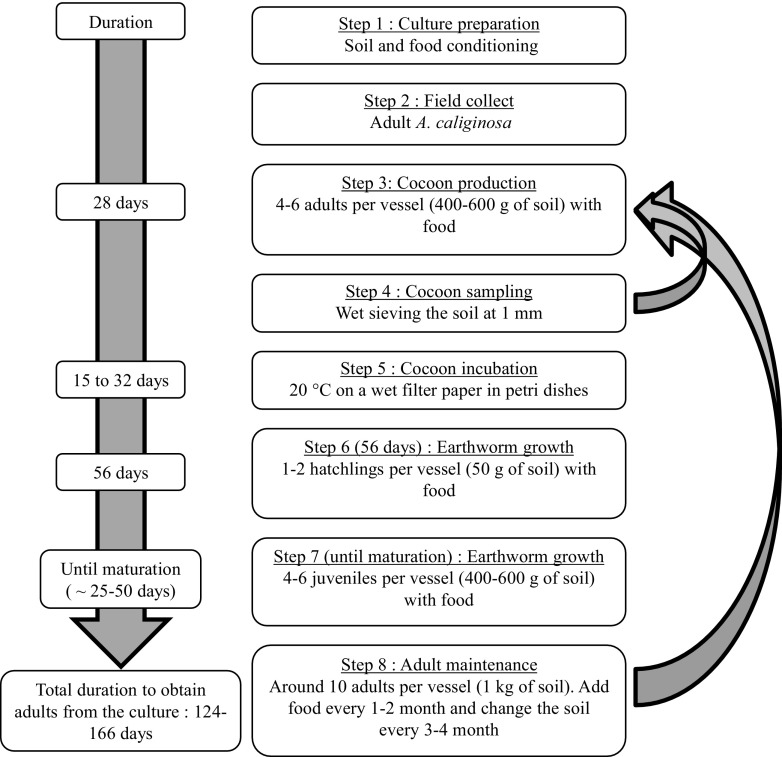
Steps and their duration for the culture of *A. caliginosa* under laboratory conditions

**Fig. 5:**
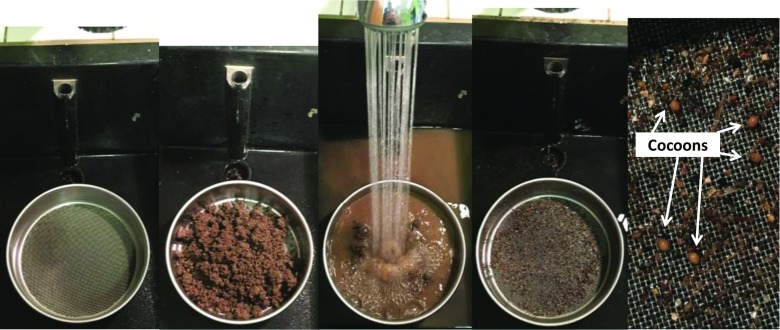
The wet sieving method for retrieving cocoons (1-mm mesh size)

The steps involved in the establishment of a laboratory culture of *A. caliginosa* have been summarized in Fig. 4. The first step is to prepare soil and food stocks as previously described. Then collect mature specimens from the field using a digging and hand-sorting method consisting of soil removal (with a spade or a fork) and searching in the upper 20 cm of the soil profile. For cocoon production (i.e., step 3), 4–6 adults can be placed in a 1-L plastic vessel, with 400–600 g of soil (dry mass) and food supply. All vessels should be sealed with a perforated (using a mounted needle) lid to allow gaseous exchange and prevent earthworms from escaping. After 1 month, individuals should be transferred into fresh substrate (food and soil). To collect cocoons, the soil should be wet sieved through a 1-mm mesh size in order to remove the soil but retain the cocoons in the sieve (Fig. 5). The collected cocoons can then be placed on wet filter paper (e.g., Whatman number 1) in petri dishes (Holmstrup et al. 1991) and incubated at 20 °C. This temperature allows a more rapid hatching than at 15 °C (Holmstrup et al. 1991) and thus optimizes hatchling production. During this period (15–32 days after cocoon collection), we recommend regular monitoring to maintain moisture level and remove hatchlings (e.g., every 2–3 days). To synchronize individuals (i.e., to get a cohort of individuals at a similar level of development), we suggest that new hatchlings are maintained (maximum 40 individuals) in a small vessel (100–200 mL, with 50 g of moist soil) at 4 °C to minimize their development until the required number of cocoons have hatched (Lofs-Holmin 1982). Then, in order to optimize growth of juveniles (step 6 in Fig. 4), it is recommended that earthworms are initially maintained individually in small vessels (e.g., 100–200 mL with 50 g of dry soil) for 56 days during which soil moisture has to be maintained. Juveniles need to be fed with 2–3 g of food per individual per month and the substrate needs to be prepared as previously described. The food should be added at the beginning (mixed with the soil). After 56 days, juveniles should be transferred to larger vessels (1 L with 400–600 g of soil), with 4 to 6 juveniles per vessel, until the development of the clitellum (at this stage individuals are sexually mature). These earthworms need to be fed with 4–6 g of food ind^−1^ month^−1^. The development of the clitellum can take between 25 and 50 days depending on soil type, food quality, and intraspecific variability. To feed individuals without changing the soil, the vessel should be emptied, earthworms removed and food mixed with the soil and returned to the vessel prior to addition of the earthworms. Soil disturbance during this procedure does not disrupt *A. caliginosa* development (personal observation).

Adult individuals can be maintained in culture for future experiments. For adult maintenance, we recommend reducing the food supply (1–2 g per individual per month is enough) to avoid excessive organic matter enrichment that could acidify the soil. Furthermore, the soil containing adults should be replaced every 3–4 months to avoid soil compaction and food accumulation which could lead to unsuitable conditions.

To produce more individuals, cocoons or hatchlings, it is also possible to go back to step 3 with adult stock. However, as suggested by Lowe and Butt (2007), we recommend avoiding the use of individuals from long-term isolated laboratory cultures. After several generations, there is a risk of inbreeding or adaptation to laboratory conditions, thus reducing the representativeness of cultured individuals compared to earthworms living in natural conditions. To avoid this situation, we recommend that laboratory cultures are renewed every 2–3 years with earthworms collected from the field.

### Experimental design for the assessment of lethal and sublethal effects of pesticides

The ISO tests can be used as a basis for the assessment of pesticide effects on *A. caliginosa.* However, we recommend adoption of the advice presented at the beginning of the “Laboratory culture and experimental design” section for soil, food type and quantity, moisture, and temperature parameters.

ISO 11268-1 (2012a) guideline can be adopted for assessment of acute toxicity of pesticides and other contaminants. The recommended density of 10 individuals per container with 500–600 g of soil (originally for *E. fetida*) is too high and 6 to 8 *A. caliginosa* individuals is proposed in this volume of soil. However, for longer exposures (i.e., more than the standard 14 days), we recommend reducing the density to 4–5 individuals per container to avoid reducing soil quality due to high earthworm activity. Furthermore, in order to avoid biomass loss during the experiment, the addition of food at a minimum rate of 3 g ind^−1^ month^−1^ is required at the beginning of the test (Bart et al. 2017). Acute toxicity tests are normally performed with adult individuals, but can be undertaken with juveniles that are potentially more sensitive to contaminants (see the “Sensitivity to pesticides” section) and change in biomass can also be monitored by weighing earthworms at the beginning and at the end of the test.

It is suggested that reproduction tests with *A. caliginosa* can follow the ISO 11268-2 (2012b) guideline with the following changes. First, a density of 4 to 5 individuals per container of 500 g dry soil is more suitable than 10 individuals as suggested in the guideline (see above). Then, we suggest using cohorts of the same age as reproduction rates decrease with age. We also propose, as suggested by Lofs-Holmin (1982) that cocoon production instead of hatchling production is assessed. Indeed, at a temperature of 15 °C, the average hatching time of cocoons is 62 days (Jensen and Holmstrup 1997) which would result in a minimum test duration of 3 months (28 days for adult exposure, and then 60–70 days for hatching). Therefore, for quicker tests, we propose directly assessing cocoon production, using the wet sieving method (see the “Development and maintenance of a laboratory culture” section). This measurement should be complemented by monitoring cocoon viability and hatching rates.

No standardized test exists for the assessment of chemical effects on earthworm growth. For a better understanding of the impact of pesticides on earthworm development, we suggest assessing this endpoint at different stages of development, from hatchlings to older juveniles (2–3 months old). In addition, we recommend assessment of maturation based on time from hatching until emergence of a fully developed clitellum (for experiments with a cohort of hatchling earthworms see the “Development and maintenance of a laboratory culture” section). To assess growth, regular (e.g., every 14 days) monitoring of individual biomass is recommended and requires earthworms to be extracted from vessels with minimal disturbance of the substrate. Extracted individuals should be weighed and put back in vessels immediately. The experiment should stop when at least 80% of the earthworms have reached maturity (i.e., with a fully developed clitellum).

Finally, *A. caliginosa* behavior in response to chemicals can be assessed by avoidance tests. From our own experience (Bart et al. 2017) and other publications (Hodge et al. 2000), the procedure described in ISO 17512-1 (2008) is suitable for *A. caliginosa*, except for the soil (see the “Laboratory culture and experimental design” section). However, as discussed in the “Sensitivity to pesticides” section, the exposure to a linear pollution gradient is a relevant alternative (Lowe et al. 2016; Brami et al. 2017b). Assessment of the capability of earthworms to detect and to avoid pesticides is relevant in risk assessment as it may more accurately reflect what happens in the field. In this situation, avoidance behavior would mean that earthworms could avoid sublethal concentrations of pesticides. Furthermore, if earthworms avoid pesticides and thus disperse from a contaminated area, they no longer provide soil ecological functions related to ecosystem services.

## Conclusion

*A. caliginosa* is one of the most dominant earthworm species in temperate agroecosystems. Its wide distribution and sensitivity to pesticides makes it a relevant soil-dwelling species for a posteriori pesticide risk assessment and more generally in soil-based ecotoxicological tests. The use of this species, along with the guidelines presented in this article, can help improve assessment of risks related to pesticide use. While *E. fetida* remains a suitable test species for initial screening of chemical effects, the use of *A. caliginosa* is relevant in complementing knowledge on the effects of pesticides, especially for pesticides already widely used on crops.

Our review has highlighted the need for further research on the impacts of pesticides on sublethal endpoints (i.e., growth and reproduction), with a focus on ecological functions such as burrowing or casting activities. Moreover, herbicides and fungicides, widely used in temperate areas, deserve more attention. This review also established that knowledge on *A. caliginosa* biology is sufficient to design laboratory experiments enabling assessment of pesticide impacts on life history traits and behavior. Data obtained from such tests could be used to understand and predict the effects of pesticides on population dynamics using modeling tools (e.g., Johnston et al. 2014).

Finally, while this review focused on *A. caliginosa*, the information provided can be used as a starting point for further research with species from *A. caliginosa* complex, i.e., *A. trapezoids*, *A. tuberculata*, *and A. nocturna.*

## Electronic supplementary material

ESM 1 (DOCX 52 kb)
