# Supplementary material for: Aporrectodea caliginosa, a relevant earthworm species for a posteriori pesticide risk assessment: current knowledge and recommendations for culture and experimental design
Source: Environ Sci Pollut Res Int. 2018 Jun 21;25(34):33867–81. doi: 10.1007/s11356-018-2579-9 (PMC6245014; doi:10.1007/s11356-018-2579-9)
Supplement: Supplementary file 1 — (DOCX 52 kb) [file 11356_2018_2579_MOESM1_ESM.docx]

**Appendix**

***Aporrectodea caliginosa,* a relevant earthworm species for a posteriori pesticide risk assessment: Current knowledge and recommendations for culture and experimental design**

Sylvain Bart^a*^, Joël Amosse^a^, Christopher N. Lowe^b^, Alexandre R. R. Péry^a^, Christian Mougin^a^, Céline Pelosi^a^

^a^ UMR ECOSYS, INRA, AgroParisTech, Université Paris-Saclay, 78026, Versailles, France

^b^ School of Forensic and Applied Sciences, University of Central Lancashire, Preston PR1 2HE, UK

*sylvain.bart@inra.fr / sylvain1.bart@outlook.fr / celine.pelosi@inra.fr

Table S1. List of references for mapping *A. caliginosa* in Fig. 1.

Table S2. Effects of metals on life history traits and behaviour of *A. caliginosa* under laboratory conditions.

For Origin: cultured (in laboratory) or collected (in the field). Method of addition: mixed (into the soil). LC50: Lethal concentration for 50% of exposed individuals. NOEC = No Observed Effect Concentration. NA= Not Available. EC_X_: Effective concentration.

Abbott I (1985) Distribution of introduced earthworms in the northern Jarrah forest of Western-Australia. Aust J Soil Res 23:263-270

Abdul G, Iffat N, Qureshi JI (2003) Earthworms of Attock District. Pak J Zool 35:171-175.

Ammer S, Ammer C (1998) Lumbricid associations on a former open clay pit after recultivation with different tree species. Forstwissenschaftliches Centralblatt 117:167-175

Baha M (1997) The earthworm fauna of Mitidja, Algeria. Trop Zool 10:247-254

Baker G, Buckerfield J, Greygardner R, Merry R, Doube B (1992) The abundance and diversity of earthworms in pasture soils in the fleurieu peninsula, south australia. Soil Biol Biochem 24:1389-1395

Baker GH, Carter PJ, Barrett VJ (1999) Influence of earthworms, *Aporrectodea spp.* (Lumbricidae), on pasture production in south-eastern Australia. Aust J Agric Res 50:1247-1257

Baker GH, Thumlert TA, Meisel LS, Carter PJ, Kilpin GP (1997) ''Earthworms Downunder'': A survey of the earthworm fauna of urban and agricultural soils in Australia. Soil Biol Biochem 29:589-597

Bart S, Laurent C, Péry ARR, Mougin C, Pelosi C (2017) Differences in sensitivity between earthworms and enchytraeids exposed to two commercial fungicides. Ecotoxicol Environ Saf 140 :177-184

Beaumelle L, Lamy I, Cheviron N, Hedde M (2014) Is there a relationship between earthworm energy reserves and metal availability after exposure to field-contaminated soils? Environ Pollut 191:182-189

Belyuchenko IS, Podarueva VI (1990) Soil mesofauna in agrocoenoses of perennial grasses and cotton in the south of Tajikistan. Doklady Vsesoyuznoi Ordena Lenina i Ordena Trudovogo Krasnogo Znameni Akademii Sel'skokhozyaistvennykh Nauk im. V.I. Lenina 29-34.

Bennour SA, Nair GA (1997) Density, biomass and vertical distribution of Aporrectodea caliginosa (Savigny 1826) (Oligochaeta, Lumbricidae) in Benghazi, Libya. Biol Fertil Soils 24:102-105

Bithell SL, Booth LH, Wratten SD, Heppelthwaite VJ (2005) Earthworm populations and association with soil parameters in organic and conventional ley pastures. Biol Agric Hortic 23:143-159.

Boag B, Palmer LF, Neilson R, Legg R, Chambers SJ (1997) Distribution, prevalence and intensity of earthworm populations in arable land and grassland in Scotland. Ann Appl Biol 130:153-165

Bouché MB (1972) Lombriciens de France. Ecologie et Systématique. INRA Ann Zool Ecol Anim Publication, France.

Brinzea G (2010) The study of lumbricidae fauna in three terrestrial ecosystems of Candesti Piedmont, Arges County (Romania). Lucrari Stiintifice - Universitatea de Stiinte Agronomice si Medicina Veterinara Bucuresti. Seria B, Horticultura: 721-728.

Cannavacciuolo M, Bellido A, Cluzeau D, Gascuel C, Trehen P (1998) A geostatistical approach to the study of earthworm distribution in grassland. Appl Soil Ecol 9:345-349

Carpene E, Andreani G, Monari M, Castellani G, Isani G (2006) Distribution of Cd, Zn, Cu and Fe among selected tissues of the earthworm (*Allolobophora caliginosa*) and Eurasian woodcock (*Scolopax rusticola*). Sci Total Environ 363:126-135

Cotton DCF, Curry JP (1982) Earthworm distribution and abundance along a mineral-peat soil transect. Soil Biol Biochem 14:211-214.

Curry JP, Doherty P, Purvis G, Schmidt O (2008) Relationships between earthworm populations and management intensity in cattle-grazed pastures in Ireland. Appl Soil Ecol 39:58-64.

Dalby PR, Baker GH, Smith SE (1998) Competition and cocoon consumption by the earthworm *Aporrectodea longa*. Appl Soil Ecol 10:127-136

Deibert EJ, Utter RA (2003) Earthworm (Lumbricidae) survey of North Dakota fields placed in the US Conservation Reserve Program. J Soil Water Conserv 58:39-45

Eijsackers H, Beneke P, Maboeta M, Louw JP, Reinecke AJ (2005) The implications of copper fungicide usage in vineyards for earthworm activity and resulting sustainable soil quality. Ecotoxicol Environ Saf 62(1):99-111

Eijsackers H, Bruggeman J, Harmsen J, de Kort T, Schakel A (2009) Colonization of PAH-contaminated dredged sediment by earthworms. Appl Soil Ecol 43:216-225

Eriksen-Hamel NS, Whalen JK (2007) Competitive interactions affect the growth of *Aporrectodea caliginosa* and *Lumbricus terrestris* (Oligochaeta : Lumbricidae) in single- and mixed-species laboratory cultures. Europ J Soil Biol 43:142-150

Ezzatpanah S, Robabeh L, Masoumeh M, Hasan S (2010) Earthworm fauna of the western Mazandaran province, Iran (Oligochaeta: Lumbricidae, Megascolecidae). Zool Middle East 67-74

Falco LB, Momo F (2010) Habitat selection by earthworms: the effect of soil cover and type. Acta Zool Mex 26:179-187

Falco LB, Sandler R, Momo F, Di Ciocco C, Saravia L, Coviella C (2015) Earthworm assemblages in different intensity of agricultural uses and their relation to edaphic variables. Peerj 3

Francis GS, Tabley FJ, Butler RC, Fraser PM (2001) The burrowing characteristics of three common earthworm species. Aust J Soil Res 39:1453-1465

Friis K, Damgaard C, Holmstrup M (2004) Sublethal soil copper concentrations increase mortality in the earthworm *Aporrectodea caliginosa* during drought. Ecotoxicol Environ Saf 57(1):65-73

Garnsey RB (1994) Seasonal activity and estivation of lumbricid earthworms in the midlands of Tasmania. Aust J Soil Res 32:1355-1367

Geras'kina AP (2009) Earthworm populations (Lumbricidae) in soils of laylands. Zool Zhurnal 88:901-906

Hendriksen NB (1991) Consumption and utilization of dung by detritivorous and geophagous earthworms in a Danish pasture. Pedobiologia 35:65-70

Holmstrup M, Petersen BF, Larsen MM (1998) Combined effects of copper, desiccation, and frost on the viability of earthworm cocoons. Environ Toxicol Chem 17(5):897-901

Holmstrup M, Costanzo JP, Lee RE (1999) Cryoprotective and osmotic responses to cold acclimation and freezing in freeze-tolerant and freeze-intolerant earthworms. J Comp Physiol B Biochem Syst Environ Physiol 169:207-214

Hong Y, Kim TH (2009) The Earthworm Composition in Agroecosystem of Sunyu Island, Korea. Korean J Environ Biol 27:135-139

Hutcheon JA, Iles DR, Kendall DA (2001) Earthworm populations in conventional and integrated farming systems in the LIFE Project (SW England) in 1990-2000. Ann Appl Biol 139:361-372.

Ivask M, Kuu A, Sizov E (2007) Abundance of earthworm species in Estonian arable soils. Eur J Soil Biol 43:S39-S42

Ivask M, Truu J, Truu M, Lohmus K, Ostonen I (2000) Earthworm Lumbricidae community in alder and aspen forest: three case studies. Balt For 6:74-77

Judas M (1990) Earthworm-zoonoses of roadside verges. Okologie und Naturschutz im Agrarraum 19:644-650

Khalil MA, AbdelLateif HM, Bayoumi BM, vanStraalen NM (1996a) Analysis of separate and combined effects of heavy metals on the growth of *Aporrectodea caliginosa* (Oligochaeta; Annelida), using the toxic unit approach. Appl Soil Ecol 4(3):213-219

Khalil MA, AbdelLateif HM, Bayoumi BM, vanStraalen NM, vanGestel CAM (1996b) Effects of metals and metal mixtures on survival and cocoon production of the earthworm *Aporrectodea caliginosa*. Pedobiologia 40(6):548-556

Khalil AM (2013) Ecotoxicological bioassays of the earthworms *Allolobophora caliginosa* Savigny and *Pheretima hawayana* Rosa treated with arsenate. Online J Biol Sci 13(3):99-105

Kherbouche D (2012) Bernhard-Reversat, F., Moali, A., Lavelle, P., The effect of crops and farming practices on earthworm communities in Soummam valley, Algeria. Eur J Soil Biol 48:17-23

Kovac L, Miklisova D, Pizl V (1996). Earthworm communities in arable soils of the East-Slovak Lowland. Rost Vyroba 42:405-410

Krivolutzkii DA, Pokarzhevskii AD, Viktorov AG (1992) Earthworm populations in soils contaminated by the chernobyl atomic power station accident, 1986–1988. Soil Biol Biochem 24:1729-1731

Lagerlof J, Goffre B, Vincent C (2002) The importance of field boundaries for earthworms (Lumbricidae) in the Swedish agricultural landscape. Agric Ecosyst Environ 89:91-103

Langdon CJ, Hodson ME, Arnold RE, Black S (2005) Survival, Pb-uptake and behaviour of three species of earthworm in Pb treated soils determined using an OECD-style toxicity test and a soil avoidance test. Environ Pollut 138(2):368-375

Ma WC (1988) Toxicity of copper to lumbricid earthworms in sandy agricultural soils amended with Cu-enriched organic waste materials. Ecological Bulletins 39:53–56

Makin AA, Miah MF, Yadav SK, Mitu D, Khan ZK (2014) Ecological diversity and abundance of earthworms in Sylhet Metropolitan area of Bangladesh. J adv botany zool 2:63-68

Makulec G (2004) Lumbricidae communities in several years old midfield shelterbelt (Turew region, western Poland). Pol J Ecol 52:173-179

Maleri R, Reinecke SA, Mesjasz-Przybylowicz J, Reinecke AJ (2007) Growth and reproduction of earthworms in ultramafic soils. Arch. Environ. Contam Toxicol 52(3):363-370

Marinissen JCY (1992) Population dynamics of earthworms in a silt loam soil under conventional and “integrated” arable farming during two years with different weather patterns. Soil Biol Biochem 24:1647-1654

Martin NA (1986) Toxicity of pesticides to allolobophora-caliginosa (Oligochaeta, Lumbricidae). New Zeal J Agr Res 29:699–706

McDaniel JP, Barbarick KA, Stromberger ME., Cranshaw W (2013) Survivability of *Aporrectodea caliginosa* in Response to Drought Stress in a Colorado Soil. Soil Sci Soc Am J 77:1667-1672

McKenzie BM, Dexter AR (1993) Size and orientation of burrows made by the earthworms *Aporrectodea rosea* and *A. caliginosa*. Geoderma 56:233-241

Mele PM, Carter MR (1999) Species abundance of earthworms in arable and pasture soils in south-eastern Australia. Appl Soil Ecol 12:129-137

Nahmani J, Capowiez Y, Lavelle P (2005) Effects of metal pollution on soil macroinvertebrate burrow systems. Biol Fertil Soils 42(1):31-39

Najar IA, Khan AB (2011) Earthworm communities of Kashmir Valley, India. Trop. Ecol. 52:151-162

Nasr HM, Ei Badawy M (2015) Biomarker Response and Biomass Toxicity of Earthworms *Aporrectodea caliginosa* Exposed to IGRs Pesticides. J Environ Analyt Toxicol 05

Nejmeddine A, WoutersTyrou D, Baert JL, Sautiere P (1997) Primary structure of a myohemerythrin-like cadmium-binding protein, isolated from a terrestrial annelid oligochaete. Comptes Rendus De L Academie Des Sciences Serie Iii-Sciences De La Vie-Life Sciences 320 :459-468

Omrani GA, Zamanzadeh M, Maleki A, Ashori Y (2005) Earthworm ecology in the Northern part of Iran: with an emphasis on compost worm *Eisenia fetida*. J Appl Sci 5:1434-1437

Owojori OJ, Reinecke AJ, Voua-Otomo P, Reinecke SA (2009) Comparative study of the effects of salinity on life-cycle parameters of four soil-dwelling species (*Folsomia candida*, *Enchytraeus doerjesi*, *Eisenia fetida* and *Aporrectodea caliginosa*). Pedobiologia 52:351-360

Pavlicek T, Csuzdi C, Nevo E (2003) Species richness and zoogeographic affinities of earthworms in the Levant. Pedobiologia 47:452-457

Perez-Losada M, Ricoy M, Marshall JC, Dominguez J (2009) Phylogenetic assessment of the earthworm *Aporrectodea caliginosa* species complex (Oligochaeta: Lumbricidae) based on mitochondrial and nuclear DNA sequences. Mol Phylogenet Evol 52:293-302

Pitkanen J, Nuutinen V (1997) Distribution and abundance of burrows formed by Lumbricus terrestris L and *Aporrectodea caliginosa* Sav in the soil profile. Soil Biol Biochem 29:463-467

Poier KR, Richter J (1992) Spatial distribution of earthworms and soil properties in an arable loess soil. Soil Biol Biochem 24:1601-1608

Qiu H, Vijver MG, Peijnenburg WJGM (2011) Interactions of cadmium and zinc impact their toxicity to the earthworm Aporrectodea caliginosa. Environ Toxicol Chem 30(9):2084-2093

Rakhmatullaev A, Gafurova L, Egamberdieva D (2010) Ecology and role of earthworms in productivity of arid soils of Uzbekistan. Dyn Soil Dyn Plant 4:72-75

Raty M, Huhta V (2004) Earthworm communities in birch stands with different origin in central Finland. Pedobiologia 48:283-291

Rosas-Medina MÁ, de León-González F, Flores-Macías A, Payán-Zelaya F, Borderas-Tordesillas F, Gutiérrez-Rodríguez F, Fragoso-González C (2010) Effect of tillage, sampling date and soil depth on earthworm population on maize monoculture with continuous stover restitutions. Soil Till Res 108:37-42

Rundgren S (2007) Lumbricidae in Iceland. Insect Systematics & Evolution 121-159.

Shekhovtsov SV, Golovanova EV, Peltek SE (2014) Invasive lumbricid earthworms of Kamchatka (Oligochaeta). Zool Stud 53

Solomou AD, Sfougaris AI, Vavoulidou EM, Csuzdi C (2012) The effects of farming practices on earthworm dynamics in olive groves of central Greece (Oligochaeta). Zool Middle East 119-126

Spurgeon DJ, Svendsen C, Rimmer VR, Hopkin SP, Weeks JM (2000) Relative sensitivity of life-cycle and biomarker responses in four earthworm species exposed to zinc. Environ Toxicol Chem 19(7):1800-1808

Sveistrup TE, Haraldsen TK, Engelstad, F (1997) Earthworm channels in cultivated clayey and loamy Norwegian soils. Soil Till Res 43:251-262

Szederjesi T, Pavlicek T, Coskun Y, Csuzdi C (2014) New earthworm records from Turkey, with description of three new species (Oligochaeta: Lumbricidae). Zootaxa 3764:555-70

Tiho S, Josens G (2000) Earthworm populations of Roosevelt Avenue (Brussels, Belgium): composition, density and biomass. Belg J Zool 130:131-138

Valckx J, Cockx L, Wauters J, Van Meirvenne M, Govers G, Hermy M, Muys B (2009) Within-field spatial distribution of earthworm populations related to species interactions and soil apparent electrical conductivity. Appl Soil Ecol 41:315-328

Van Rhee JA (1975) Copper contamination effects on earthworms by disposal pig waste in pastures. In : Vanek (Ed.). Progress in Soil Zoology. Proc. 5^th^ Int. Colloq Soil Zool Prague. 1975. Pp. 451-457

Yu YL, Wu XM, Li SN, Fang H, Tan YJ, Yu JQ (2005) Bioavailability of butachlor and myclobutanil residues in soil to earthworms. Chemosphere 59:961-967

Zorn MI, Van Gestel, CAM, Eijsackers H (2005) Species-specific earthworm population responses in relation to flooding dynamics in a Dutch floodplain soil. Pedobiologia 49:189-198
